# Drop-on-demand printing of amine-responsive fluorescence-ratiometric sensor array for online monitoring meat freshness

**DOI:** 10.1016/j.fochx.2024.102099

**Published:** 2024-12-18

**Authors:** Zhijian Wang, Xudong Shi, Jingze Guo, Lin Wang, Meilin Cao, Shiyao Wang, Yisheng Chen

**Affiliations:** College of Food Science and Engineering, Shanxi Agricultural University, Taiyuan, Shanxi, China

**Keywords:** Fluorescence-ratiometric sensor array, Smart printing, Meat freshness

## Abstract

Aiming to enable online freshness-monitoring of meat within modified-atmosphere package, we developed a ratiometric array that was fluorescently responsive to volatile organic compounds-ammonia (NH_3_) released by protein decaying. The array was consisted of two 3 mm × 6 mm rectangles precisely and uniformly printed with fluorescein isothiocyanate (FITC) as indicator and rhodamine B (RhB) as internal reference on the filter-paper, respectively. The fluorescence intensity of the array area was calibrated according to Green/Red ratio of the digitalized pixels extracted from images facilitated by a smartphone. The fluorescence-ratiometric sensor array displayed remarkable detection performances, including high sensitivity (LOD = 1.1 ppm), stability (91 % responding attenuation over 10 d of storage) and reproducibility (RSD < 10 %), which was further validated with real pork and shrimp samples. Subsequently, the fluorescent signals of the dual-rectangle array showed high correlation to the total volatile base nitrogen value that was officially used for indexing the meat freshness status.

## Highlights

• Fluorescence-ratiometric sensor array was prepared using drop-on-demand printing.
• The sensor array showed good sensitivity to the VOCs associated with meat spoilage.
• The sensor array displayed excellent reproducibility and storage stability.
• The performance of the established sensor array was validated with real meat samples.

## Graphical abstract

**Figure:**
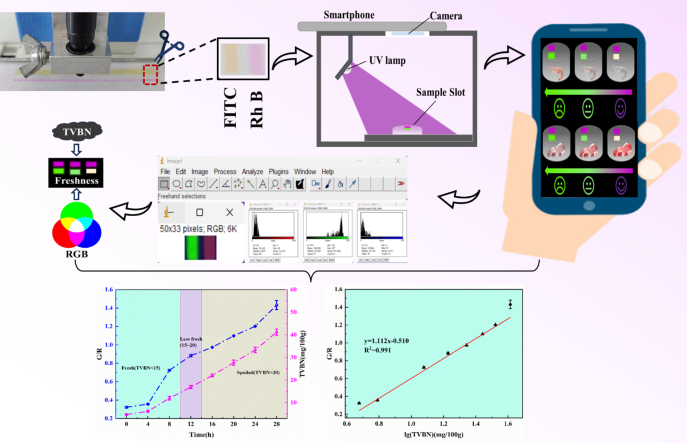

## 1 Introduction

In nowadays, modified atmosphere packaging was becoming increasingly popular and indispensable for industrialized logistic and marketing of livestock and aquatic products, in order to preserve the freshness and prolong the shelf-life. Fresh meat, including beef, pork and shrimp, contained high-quality dietary protein, water, trace elements, vitamins and balanced amino acids (González, Marquès, Nadal, & Domingo, 2020). Meat was prone to spoil under ambient conditions due to its nutritional characteristics, releasing volatile amines, like ammonia and biogenic amies. It also was the main reason for the decrease of meat freshness. Therefore, concentrations of these VOCs associated with meat spoilage would be the ideal marker of meat freshness, as an important guiding factor for consumption (Yuanming, Jinfeng, & Jing, 2024). However, the official method using the value of total volatile basic nitrogen (TVBN) to index the freshness status of meat suffered several intrinsic difficulties, for example, instrument-dependence and time-consuming operation(Zou et al., 2024). Worse still, meat sealed in the package cannot be sampled for measurement in this way. With this regard, simple, fast, cost-effective and reliable freshness-senor system able to be easily incorporated into the package material had attracted marked attention, world-widely(Shao, Lan, & Xie, 2024).

Optical artificial-scent sensor array (OASSA) was a bionic system enlightened by human olfactory system (Zhang, Zhang, Mujumdar, & Wang, 2024). These sensors were spectrally responsive to certain volatile molecules. In addition, sensor array analysis may take advantages of multi-channel, multi-dimensional, and simultaneous information acquisition by eye-inspection and image documentation(Xiaowei et al., 2024). The sensor array can sensitively detect the change of the odor of the test sample and simulate the function of the animal nose. Compared with other advanced methods, it had a relatively low equipment cost (Yang et al., 2021). Zhang et al. (Zhang et al., 2023) developed a sensor array by screening color-sensitive materials and using RGB difference to achieve portable and intelligent pork freshness detection. Therefore, OASSA was opening a new horizon for in-situ evaluation of meat freshness, as an ideal tool for visualized flavor perception(Romruen, Karbowiak, Auras, & Rawdkuen, 2024).

According to the optical types of signals, OASSA can be divided into colorimetric sensor array (CSA)(Yuandong, Ji, Jun-Hu, & Da-Wen, 2024) and fluorescence sensor array (FSA)(Jaguey-Hernández et al., 2021). CSA relied on color changes to detect analytes, often susceptible to background stray light interference (Ma et al., 2023). In contrast, the FSA was based on the detection of the fluorescence signal generated by photons released when excited molecules returned to the ground state. This signal generation and detection process was highly selective. Since the fluorescence signal intensity was proportional to the target molecule concentration, FSA provided a clear and reliable detection signal even in complex samples (Ding et al., 2024). Therefore, FSA effectively distinguished the target molecule's fluorescence signal even with background stray light, remarkably increasing the detection sensitivity and anti-interference capability. Cai et al. (Cai et al., 2022) prepared a multicolor FSA based on three dyes to monitor the freshness of shrimp. Each sensing label showed good performance in optical properties, NH_3_ sensitivity and fluorescence discoloration pathways.

For an activated luminescence, the fluorescence intensity showed only slight changes. The limited sensitivity of eye-inspection on these changes could increase experimental error (Magnaghi, Alberti, Quadrelli, & Biesuz, 2020). This limitation might even render the naked-eye detection mode ineffective. However, the fluorescence-ratio sensor array (FRSA) can eliminate interference from uneven concentration, temperature, and other factors by self-calibrating the signal intensity of two different fluorescence emission peaks. This allowed for accurate determination of analyte content. Jia et al. (Jia, Tian, Bai, & Zhang, 2019) developed a FRSA utilizing protoporphyrin and fluorescein isothiocyanate-modified cellulose for the real-time visual assessment of seafood freshness. The probe exhibited a fluorescence color shift to green and yellow after 1 d storage at 25 °C and 4 °C, respectively. After 5 d at −16 °C, the fluorescence color turned yellow. Nevertheless, FRSA faced issues like complex material preparation and low fluorescence utilization efficiency. Additionally, the lack of intelligent result display and challenges in mass production limited their practical applications.

Inkjet printing was a downstream processing technology applied in the food packaging industry. However, inkjet printing technology couldn't precisely print the volume of the printed image, therefore ensuring a stable color block was difficult (Wang et al., 2024). Furthermore, it was complex to quantitatively analyze the correlation between the color variation of sensor and the chemical index of food spoilage. Additionally, cleaning the printhead of the inkjet printer was not easy, and it might become clogged if not used frequently. Therefore, there was an urgent need to design a smarting printing device that could be easily cleaned and used to print corrosive liquids in microliter quantities.

Fluorescein isothiocyanate (FITC) exhibited green fluorescence, pH sensitivity, good stability and biocompatibility. Recent studies evidenced that FITC-based films were an ideal tool for monitoring the freshness of shrimp. Rhodamine B (RhB) emitting red fluorescence has the advantages of low-cost fluorescence quantum yield, high absorption coefficient. What's more, large fluorescence is almost unaffected by pH value, and is widely used as a reference signal for ratio fluorescence intelligent packaging(Tao et al., 2022). Therefore, this study designed and fabricated a paper-based FRSA modified with RhB and FITC using smart printing technology. The initial fluorescence intensity was fine-tuned by optimizing the FITC concentration. Then, images of FRSA were easily documented with a smart phone for chromaticity analysis. Under optimized conditions, the performance of the FRSA, including stability, reversibility, and reproducibility was evaluated, benchmarking with the official standard method.

## 2 Materials and methods

### 2.1 Materials

Fluorescein isothiocyanate (FITC) and rhodamine B (RhB) were purchased from Aladdin Biochemical Technology Co., Ltd. (Shanghai, China). Ammonia solution (NH_3_•H_2_O, 25 % aqueous solution) was purchased from Shanghai Aladdin Biochemical Technology Co., Ltd. Ethanol, magnesium oxide (MgO), boric acid, hydrochloric acid (HCl) was purchased from Shanghai McLean Biochemical Co., Ltd. All reagents and materials were analytical reagent grade and can be used without further purification. All the experimental water was ultrapure water. Substrate qualitative filter paper for the preparation of the FRSA was purchased from Hangzhou Special Paper Co., Ltd. (Hangzhou, China), Model 102. Fresh pork (pork ribs) and shrimp were purchased from Taiyuan Xiaodian Farmers' Market.

### 2.2 Preparation of the FRSA

1 mg/mL FITC and RhB anhydrous ethanol solution were prepared respectively. Subsequently, the fluorescent dyes were precisely and non-contactly deposited on 10 cm × 9 cm ordinary filter paper with nitrogen as the carrier and Bio-CHEMSPRAY-I (Xuaming Intelligent Equipment Co., Ltd., Suzhou, China) according to the strip (60 mm × 3 mm). Printing different dyes was controlled by increasing the spacing of y-axis strips. It is worth noting that one batch can be cut into ten 6 mm × 3 mm sensing areas, and the preparation can be completed in 3 min. The specific parameters of printing were as follows: the initial distance from the bottom of the strip is 5 mm, the left end is 5 mm, the distance between x-axis strips is 10 mm, the distance between y-axis strips is 4 mm, the spotting speed (Zv) is 0.1 mm/s, the moving speed of X-axis (Xv) is 5 mm/s, and the single moving step of Y-axis is 1 mm. When used, the prepared fluorescent colorimetric sensing array was pasted on the top of the container cover with commercially available transparent tape, and the sensing area was upward and attached to the inside.

### 2.3 Color responsiveness to NH_3_

Determination of gases was carried out in conventional Petri dishes (100 × 15 mm) at room temperature. The sensor was attached to the lid. A given volume of liquid analyte (NH_3_) was filled into the dish. The desired gas concentration was reached by covering the lid and sealing it with parafilm. The required ammonia concentration (5, 25, 50, 250 and 500 ppm) is obtained by injecting a specific volume of ammonia solution into the container. The resulting amine gas concentration (C_ppm_) is calculated using the following formula (1)(Xu, Wang, Ding, Zhou, & Ding, 2024):

(1) $$C_{\mathrm{ppm}}=\frac{V_{\mathrm{\mu L}}\times D_{\mathrm{mg}/L}\times W}{M_{g/\mathrm{moL}}\times V_{L}}\times 22.4\times 10^{-9}$$

where *V*_μL_ is the amine solution volume, *D*_*mg/L*_ is the amine solution density, *W* is the mass fraction of the amine solution, *M*_*g/mol*_ is the molecular weight of the amine solution, and *V*_*L*_ is the volume of Petri dish.

After that, the prepared FRSA was immediately placed on the top of the container. The 20 W 365 nm ultraviolet lamp with the portable dark box for shooting (equipped with smart phone shooting hole position) (WFH-203, Yixin, Shanghai, China) was used as the excitation light source for the FRSA label. The change of G/(R + G + B) value was analyzed by mobile phone photo measurement. This method was employed to investigate the response of fluorescein isothiocyanate to different concentrations of ammonia. The response of rhodamine B to different concentrations of ammonia was investigated according to the above method. The change of R/(R + G + B) value was analyzed by mobile phone photo measurement.

### 2.4 Optimization of FRSA conditions

#### 2.4.1 Determination of response time to ammonia

It took a certain time for the FRSA to react with ammonia. If the response time was too short, the stability of the FRSA signal was poor, which seriously affected the subsequent analysis. If the response time is too long, the FRSA signal will be oversaturated. This can lead to the loss of important signal mutation information and result in a waste of time (Song et al., 2023). Therefore, this study investigated the response of the FRSA at different times of 10, 20, 30, 40 and 50 min at room temperature, and analyzed the change of G/R value. Determine the optimal time for the reaction of the FRSA with ammonia.

#### 2.4.2 Fluorescein concentration optimization

To obtain the best detection performance, the addition rate of RhB and FITC was optimized. Under 365 nm ultraviolet light at room temperature, the color response of FITC/RhB (1 mg/mL) indicator card with different FITC concentrations (0.15, 0.2, 0.25, 0.3, 0.35, 0.4, 0.45 mg/mL) in 500 ppm ammonia was observed. The change of G/R with the addition of FITC was determined.

### 2.5 Performance measurement of the FRSA

#### 2.5.1 Stability

In order to investigate the stability of the fluorescent test paper to air, we placed the FRSA fluorescent test paper in a closed headspace bottle, and then heated the headspace bottle in an oven at 50 °C. A headspace bottle was taken out at 0,0.5,1.0,1.5,2.0,2.5 h, and the change of G/R was determined.

To evaluate the color stability of the FRSA during storage, the fluorescence indicator card was placed at room temperature (25 °C, 50 % RH) for 10 d. The color parameter RGB value of the FRSA was recorded every 2 d(Z. Yang, Chen, & Wei, 2024). The 0 d FRSA was used as the color reference to determine the change of G/R with the extension of storage time.

#### 2.5.2 Reversibility

In order to evaluate the reversibility of the FRSA, the FRSA exposed to ammonia environment for 40 min was heated in an oven at 50 °C. The color change was recorded with a mobile phone at 0, 0.5, 1.0, 1.5, 2.0 and 2.5 h, respectively(). This was done to determine the change of G/R with the extension of heating time.

#### 2.5.3 Reproducibility

Print three batches of FRSA measurements separately, or expose to three batches of pork and shrimp purchased separately. The repeatability of array printing was evaluated by measuring the G/R average value three times. For comparison, the repeatability of the source meat was evaluated from three separate purchases, but measured using an array of single print batches.

### 2.6 The application of FRSA in the freshness monitoring of meat samples

In this experiment, shrimp and pork were selected as actual samples to verify the practicability of the FRSA. Refer to the method of Liu et al. (Liu et al., 2022). Made some modifications, as follows: Fresh shrimp and pork purchased from supermarkets were segmented, and 10 ± 0.5 g of shrimp and pork were accurately weighed and placed in a petri dish. FRSA was pasted on top of the petri dish and not in contact with the sample to ensure that the petri dish was sealed without leakage. After that, shrimp and pork were stored in a constant temperature incubator at 25 °C for 28 h and 56 h, respectively. The fluorescence change of the FRSA was recorded by mobile phone, and the freshness of the sample was analyzed by RGB results.

### 2.7 Verification of the detection accuracy of the FRSA

For the purpose of measuring the detection accuracy of the FRSA, the TVBN of shrimp and pork stored at 25 °C was detected. The specific measurement method was based on all measurements were repeated 3 times to take the average. The TVBN value is estimated according to the method of Pan et al. (Pan et al., 2023). 10 g samples were put into a conical flask containing 100 mL of distilled water, and the samples were shaken for 30 min. Then, 5 mL 10 g/L magnesium oxide was added to 10 mL solution filtered by filter paper and distilled for 5 min using a semi-automatic Kjeldahl nitrogen analyzer. 20 g/L boric acid 10 mL absorption distillate was titrated to purple with 50 μL mixed indicator solution (1 g/L methyl red ethanol solution: 1 g/L bromocresol green ethanol solution = 1:5) and titrated to colorless with 0.1 mol/L hydrochloric acid. All measurements were repeated three times in mg/100 g.

### 2.8 Image acquisition preprocessing and color feature extraction

The Image J software was used to preprocess the image and extract the color features. Image preprocessing primarily involved despeckling to remove noise, retaining sharp edges, enhancing local contrast, normalizing with a median filter, and detecting regions of interest (ROI) using a threshold method. Since the color of each sensing area was very different from the background color (black), the ROI area can be easily identified using the contour detection function in Image J software. This ensured that the position of the ROI area in the FRSA remained consistent during storage. Among them, speckle elimination, edge sharpening and contrast enhancement were mainly realized by the functions of Remove Outilers, Despeckle, Mean Filters and Enhance Contrast in Image J software. After image preprocessing, the RGB color space was explored using Image J software. The extraction and calculation of color features referred to a previous study (Y. Lin, Ma, Sun, Cheng, & Wang, 2023).

### 2.9 Statistical analysis

All experiments were repeated triplicate to calculate their average values. A one-way analysis of variance (ANOVA) followed by Duncan's multiple range test was used to separate the significant differences (*P* < 0.05) between groups.

## 3 Results and discussion

### 3.1 Construction of the FRSA

**Fig. 1:**
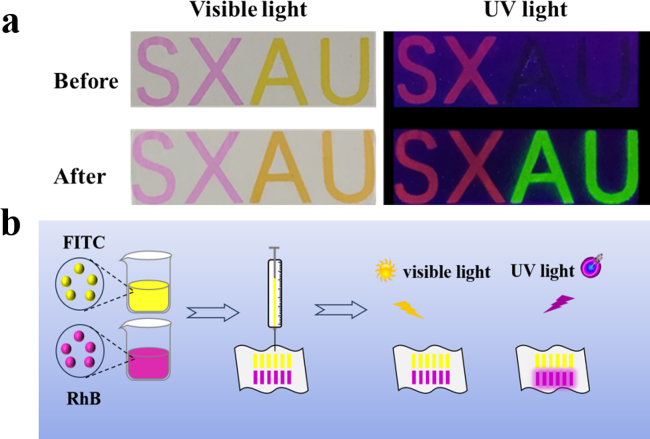
(a) Pattern designability of programmable spraying. The fluorescence images of fluorescein isothiocyanate and rhodamine B before and after reaction with ammonia under visible light and 365 nm ultraviolet light. (b) Preparation of the FRSA.

Conventionally, the array was spotted by capillary and pipette, accompanied by uncontrollable band size and diffusion. These issues leaded to poor consistency in the prepared fluorescence sensor. With the increase of the number of samples, the possibility of mutual contamination between adjacent sample points increased (Li et al., 2024; Liu et al., 2024). Driven by this, a lot of efforts have focused on the development of effective preparation methods. Based on computer software programming, the liquid micro-jet could be precisely controlled. The method of layer-by-layer controllable deposition of microfluidics, such as small molecules, macromolecules and micro-nano materials, can achieve this purpose well through this precise control(Zou et al., 2024). As shown in Fig. 1a, by smart printing, the FRSA prepared can efficiently obtain a sensing unit with uniform size and color. As shown in Fig. 1b, the FRSA prepared by programmable spraying technology can accurately control the deposition position and shape of the sensing material, achieve high-precision patterning design, and greatly improve the specificity and selectivity of the sensor array. The programmable spraying process is efficient and controllable, which is conducive to large-scale production and significantly reduces the production cost. It provides a solid foundation for the wide application of fluorescent ratio sensor arrays in many fields.

### 3.2 Measuring ammonia use the FRSA

During meat storage, tissue enzymes, external microorganisms and other factors caused changes in its composition. It leaded to the deterioration and decay of the meat. The process produced short-chain alcohols, biogenic amines (such as ammonia and trimethylamine), sulfur-containing volatile substances, etc. These volatile compounds can be used as markers to indicate the freshness and spoilage stage of meat (Kang et al., 2018). Here, we tested the response of the FRSA to the characteristic volatile gas ammonia (NH_3_) related to meat freshness. In general, the principle of selecting chemically responsive dyes was based on whether they contain interaction centers that can interact strongly with the analyte.

**Fig. 2:**
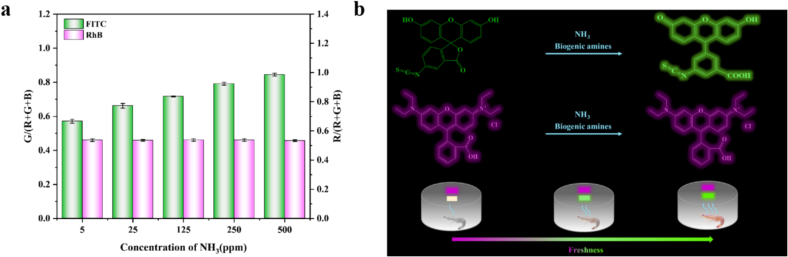
(a) Potential detection mechanism of the FRSA. (b) Mechanism verification.

The compact and lightweight smartphone contained numerous embedded functions and reliable modules (Yu et al., 2023). Through customized accessory devices, it is helpful for the integration, operation simplification and information sharing of real-time detection devices. The image acquisition and data processing functions of smart phones can assist the detection of the FRSA to improve portability and detection accuracy (Li, Xu, Peng, & Wang, 2024). As shown in Fig. 2a, the FRSA consisted of fluorescein isothiocyanate and rhodamine B. For fluorescein isothiocyanate, when exposed to NH_3_, the fluorescence of fluorescein isothiocyanate changed. With the increase of NH_3_ concentration, the fluorescence intensity G/(R + G + B) of FITC gradually increased. This is because the lactone ring on FITC was opened under the action of ammonia to form a carboxyl group. Under alkaline conditions, the phenolic hydroxyl group was converted into an unsaturated six-membered ring ketone through an acylation reaction (Quan et al., 2021). The reaction process was shown in Fig. 2b. This caused the FRSA to change color in response to NH_3_, enhancing NH_3_ detection efficiency. The R/(R + G + B) of rhodamine B remained unchanged, indicating it does not respond to NH_3_ and can serve as a reference signal. A similar observation was reported for the Zein/RhB-Flu film by Liu(Liu, Jiang, et al., 2022). Thus, the sensing array comprised FITC and RhB, and its fluorescence intensity can be adjusted based on the G/R ratio change.

### 3.3 Response time of the FRSA to ammonia

The reaction time of the analyte with ammonia is a key factor in the FRSA. Studies have shown that the reaction time significantly impacts the stability of FRSA signals and the accuracy of analysis results. Specifically, if the reaction time is too short, the signal stability is insufficient, which adversely affects the subsequent analysis (Fernandes et al., 2020). On the contrary, if the reaction time is too long, the signal will be oversaturated, resulting in the loss of key signal mutation information and the waste of time (Bai et al., 2024). Therefore, this study aimed to optimize the response time of the FRSA to ammonia.

**Fig. 3:**
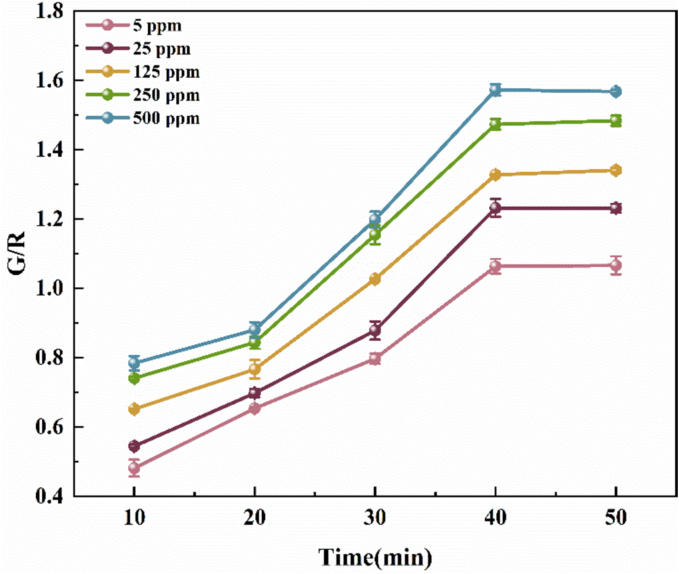
Determination of the FRSA and ammonia reaction time.

The response time at different time points at room temperature was used as a variable in the experimental design. The range was set from 0 to 50 min, and the time interval was 10 min. A total of 6 sample points was collected. Fig. 3 showed the response of the FRSA to different concentrations of NH_3_ gas with time. Over time, the FRSA in ammonia gas showed a gradual increase in the G/R value, stabilizing after 40 min. At this time, there was a positive correlation between ammonia concentration and G/R value. Thus, the response time was set to 40 min for subsequent colorimetric determination.

### 3.4 FRSA fluorescein concentration optimization

**Fig. 4:**
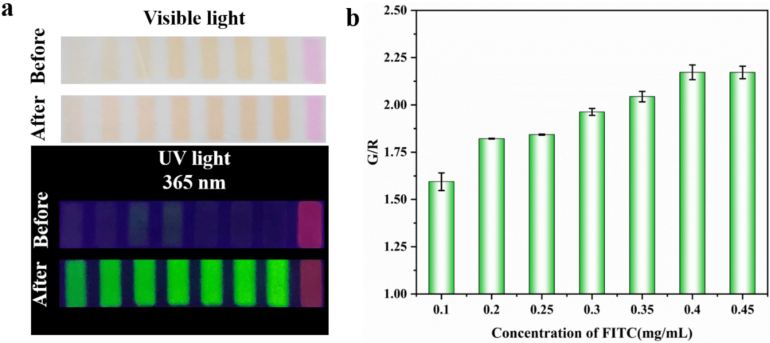
Optimization of fluorescein isothiocyanate concentration of the FRSA under (a) visible light and (b) 365 nm UV light irradiation.

The FITC in the FRSA, as a response substance to NH_3_, showed excellent response to NH_3_. Therefore, we optimized the concentration of FITC. As shown in Fig. 4a, under white light conditions, the yellow color of FRSA intensified with increasing fluorescein concentration at 500 ppm ammonia. However, after reacting with ammonia, the FRSA turned orange, with no significant color difference between the two states. Under 365 nm UV light, the green fluorescence of FRSA was gradually enhanced. As shown in Fig. 4b, When the concentration of fluorescein was 0.4 mg/mL, the green fluorescence of FRSA reached saturation, and the G/R value tended to be stable. Considering the color change of the FRSA before and after the reaction under sunlight and ultraviolet light, 0.4 mg/mL was determined as the optimal FITC dose for the preparation of the FRSA.

### 3.5 Determination of ammonia sensitivity of the FRSA

**Fig. 5:**
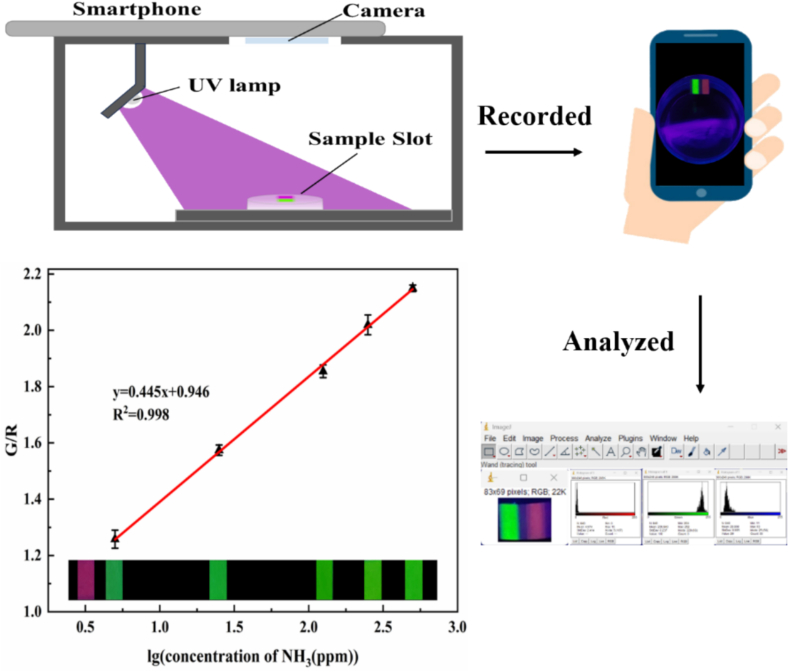
Linear relationship between G/R value of the FRSA and ammonia concentration.

As shown in Fig. 5, the response of the FRSA to different concentrations of NH_3_ was studied. The fluorescence intensity ratio G/R of the FITC and RhB sensing arrays showed a strong linear correlation (R^2^ = 0.998) with the logarithmic ammonia concentration from 5.0 ppm to 500 ppm. The LOD for NH_3_ was 1.1 ppm (Sa/b = 3). It showed that the concentration of ammonia can be quantitatively detected by the fluorescence color G/R value, revealing that FRSA combined with the smartphone can be used for the determination of ammonia level. Thus, the FRSA with FITC and RhB can serve as a quick, easy, and quantitative method for detecting ammonia. It can also be used as a smart label on packaging to monitor shrimp and pork freshness, making it ideal for on-site non-destructive visual food freshness detection.

Significantly, the FRSA not only has a wide linear range and low LOD, but also the two-color ratio fluorescent probe can achieve dynamic color conversion, which greatly improves the sensitivity of visual detection. Moreover, the prepared paper-based sensor as an excellent system for sensing meat freshness, making it more advantageous and versatile than powder and hydrogel sensors(Zhang, Sun, et al., 2023; Zhou, Li, He, & Hou, 2024).

### 3.6 Stability and reversibility of the FRSA

**Fig. 6:**
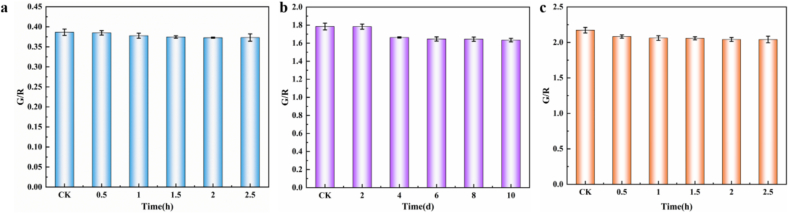
(a) Air stability, (b) storage stability and (c) irreversibility of the FRSA.

As shown in Fig. 6a, the G/R of the FRSA changed little with the extension of heating time. Even after heating for 2.5 h, the G/R of the FRSA can still maintain more than 95 %, indicating that the fluorescence ratio sensing array had good stability in air. As presented in Fig. 6b, the G/R of the FRSA changed little with the extension of storage time. Even after 10 d of storage, the fluorescence intensity of the FRSA retained over 91 % of its fluorescence intensity, demonstrating excellent storage stability. As shown in Fig. 6c, the fluorescence intensity of the the FRSA after reaction in 500 ppm ammonia environment can still maintain 94 % even after heating for 2.5 h at 50 °C, without returning to the original state. This made the FRSA is suitable for one-time use. Hence the indicator can still show freshness even if the packaging is compromised during transport and storage, in real-world packaging scenarios.

### 3.7 Freshness monitoring of the FRSA in real samples

**Fig. 7:**
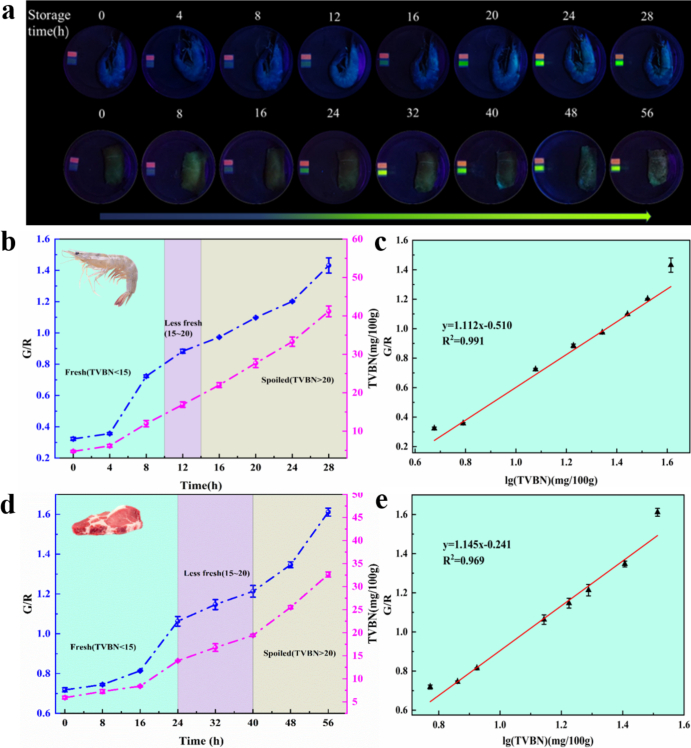
(a) Freshness of shrimp and pork at 25 °C (image under 365 nm ultraviolet light). (b,d) Fluorescence (G/R) values of shrimp and pork at different storage times at 25 °C and TVBN values of shrimp and pork. (c,e) The linear relationship between the fluorescence (G/R) value and the lg(TVBN) value of shrimp and pork at 25 °C.

When fresh shrimp and pork were stored at 25 °C for 0 h, the FRSA showed bright pink fluorescence in Fig. 7a. As storage time increased, the green fluorescence of the FRSA gradually increased, indicating that shrimp and pork had begun to spoil and produce biogenic amines. When the shrimp was stored at 25 °C for 12 h, it began to show light green fluorescence. With the increase of storage time, the green fluorescence of the test paper gradually increased. After 24 h at 25 °C, shrimp exhibited bright green fluorescence. Pork stored under the same conditions showed light green fluorescence. As storage time increased, the green fluorescence intensified. After 48 h at 25 °C, pork also exhibited bright green fluorescence.

### 3.8 Verification of the detection accuracy of the FRSA

To validate the effectiveness of the FRSA, we compared its results with the TVBN standard reference method commonly used by the Food Inspection Administration. According to national standards, the TVBN value for fresh meat should be ≤15 mg/100 g. Meat products with TVBN values between 15 and 20 mg/100 g are considered sub-fresh, while values >20 mg/100 g indicate spoilage. Fig. 7b and Fig. 7d showed that the TVBN content in shrimp and pork increased over time during storage. Initially, the TVBN values were 4.74 mg/100 g for shrimp and 5.91 mg/100 g for pork. After 12 h of storage, the TVBN value for shrimp reached 16.91 mg/100 g. For pork, after 32 h of storage, the TVBN value reached 16.78 mg/100 g, exceeding the fresh meat threshold. At this stage, shrimp and pork were sub-fresh state. The TVBN value of shrimp stored for 16 h was 21.99 mg/100 g, and for pork stored for 48 h, it was 25.52 mg/100 g, both exceeding 20 mg/100 g, showing spoilage and inedibility. Accordingly, based on the TVBN value standard for meat products, shrimp can be considered fresh within 0–12 h of storage, sub-fresh within 12–16 h, and spoiled after 16 h. Pork remained fresh within 0–32 h, sub-fresh within 32–48 h, and spoiled after 48 h.

Fig. 7c and Fig. 7e illustrated the correlation between the G/R value from the FRSA and the TVBN content in meat stored at 25 °C, showing similar trends. Hence, the FRSA can serve as a straight forward alternative for assessing meat freshness through TVBN levels.

### 3.9 Reproducibility of the FRSA

With the aim of assess the reproducibility of the FRSA, we used three batches of different sensor arrays to measure meat products three times consecutively. Additionally, shrimp and pork purchased at three different times were tested using the same batch of sensors for comparison. In all reproducibility studies, 10 g of each meat was stored at room temperature for 48 h, with three parallel samples measured per group. The reproducibility of the array was evaluated by the average G/R values of the three tests. As shown in Table 1, the G/R values of shrimp and pork purchased in a single purchase were 1.77 ± 0.08 and 1.80 ± 0.03, measured by three printed batches of the array. The reproducibility of meat was evaluated by three separate purchases, using a single print batch array. The G/R values for shrimp and pork were 2.22 ± 0.16 and 2.32 ± 0.14, RSD < 10 %. This indicates that the array demonstrates good reproducibility and excellent repeatability in detecting volatile gases in spoiled meat.

**Table 1:** Reproducibility of sensor array response between different batches of sensor array and meat products.

	**Different arrays**		**Different samples**	
	**(*n*** **=** **3)**		**(n** **=** **3)**	
	Average G/R	RSD (%)	Average G/R	RSD (%)
Shrimp	1.77 ± 0.08	5.5	2.22 ± 0.16	9.3
Images				
Pork	1.80 ± 0.03	2.0	2.32 ± 0.14	7.5
Images				

## 4 Conclusions

To conclude, a FRSA was constructed via drop-on-demand printing technology to realize visual monitoring of meat freshness. Owing to the stimulus-response structural change, FITC undergoes a sensitive fluorescence enhancement in response to alkaline amines, while RhB displays an intrinsic control effect. Significantly, the obtained FRSA displayed remarkable sensitivity (LOD of ammonia at 1.1 ppm) and reproducibility (RSD < 10 %). Furthermore, after 10 days of storage, the FRSA retained over 91 % of its fluorescence intensity, demonstrating excellent storage stability. As a simple, low-cost, high-contrast and fast-response smart trademark, the FRSA was successfully applied for visually monitoring the freshness of shrimp and pork. Validation against the standard TVBN freshness evaluation method confirms the accuracy and reliability of this sensor. However, this method is difficult to achieve rapid discrimination and accurate quantification of different biogenic amines. In the future, the integration of machine learning techniques may provide the possibility to further classify and quantify different amine markers for monitoring meat freshness, and this new strategy may be extended to explore food spoilage monitoring methods for other types of foods.

## CRediT authorship contribution statement

**Zhijian Wang:** Writing – original draft, Validation, Software, Data curation. **Xudong Shi:** Software, Conceptualization. **Jingze Guo:** Data curation. **Lin Wang:** Writing – original draft. **Meilin Cao:** Methodology. **Shiyao Wang:** Supervision, Investigation. **Yisheng Chen:** Writing – review & editing, Supervision, Resources, Funding acquisition, Conceptualization.

## Declaration of competing interest

The authors declare that they have no known competing financial interests or personal relationships that could have appeared to influence the work reported in this paper.

## Data Availability

The authors are unable or have chosen not to specify which data has been used.
